# Applications and challenges of utilizing digital pathology and AI-enabled workflows in clinical trials

**DOI:** 10.1016/j.jpi.2025.100542

**Published:** 2026-01-02

**Authors:** Manu Sebastian, Harsh Batra, Monika Lamba Saini, Staci Kearney, Lorcan Sherry, Serge Alexanian, Michael Cohen, William Weber, Joe Lennerz, Anil V. Parwani

**Affiliations:** a The University of Texas MD Anderson Cancer Center, Houston, TX 77030, United States of America; b McMaster University, Hamilton L8S 4L8, Canada; c ADC Therapeutics, Imperial College, White City Campus, London W12 0BZ, UK; d Elevation Strategic Development, 7314 Rossman Gulch Rd, Morrison, CO 8046, United States of America; e OracleBio Limited, Biocity Scotland, North Lanarkshire ML1 5UH, UK; f Pictor Labs, Los Angeles, CA 90064, United States of America; g Wake Forest University School of Medicine, Bowman Gray Center for Medical Education, 475 Vine Street, Winston-Salem, NC 27101, United States of America; h Takeda, 650 E Kendall St, Cambridge, MA 02142, United States of America; i BostonGene, University Office Park III, 95 Sawyer Road, Waltham, MA 02453, United States of America; j Wexner Medical Center, Ohio State University, 410 W 10th Ave, Columbus, OH 43210, United States of America

**Keywords:** Digital pathology, Clinical trials, Artificial intelligence, Translational medicine, Precision medicine, Regulations

## Abstract

This is a comprehensive review on current utilization and challenges of digital pathology adoption in clinical trials and aims to provide a broad view on its impact on pathology review processes in clinical trials. It provides an overview of current pathology review practices in clinical trials and unique advantages digital pathology adoption can offer. The key areas including existing workflows, use case scenarios in different disease areas in clinical trials, including but not limited to patient identification and pre-screening, and regulatory aspects have been described with relevance. In addition, the review delves into the integration of genomics, AI, image analysis, radiology, and advanced computational pathology, to propose measures to enhance clinical trial outcomes. The current regulatory landscape around digital pathology adoption and potential future advancements in this field are also discussed as appropriate.

## Highlights

• A comprehensive review of digital pathology in clinical trials.
• An outline of advantages to adopt digital pathology in clinical trials in today's data driven landscape.
• Discussion of AI and computational pathology practical applications.
• Addressing of regulatory aspects of digital pathology use in clinical trial setting.
• Brief about future integrated approaches.

## Introduction and overview

Clinical trials are studies involving human volunteers for evaluating new treatments through sequential phases to identify safer, more effective methods of treating diseases using new drugs or devices or new combinations of existing drugs. These sequential phases include phase I through phase 3 (phase 1 focuses on safety, phase 2 focuses on efficacy, and phase 3 focuses on comparison with existing treatment) for regulatory approval and phase 4 (focuses on long-term effects), which follows regulatory approval for tracking long-term safety. Pathology plays a critical role in all phases, including patient recruitment, eligibility, enhanced detection and grading of disease, efficacy and response monitoring, safety evaluation, predicting endpoints, and pre-clinical research.

The role of pathology in clinical trials has become more critical with the use of tissue biomarkers in various targeted therapies. In clinical trials, routine hematoxylin and eosin (H&E) staining, immunohistochemistry (IHC), in-situ hybridization, and multiple molecular profiling tests are used frequently and are central to the process. Digital pathology (DP) adoption for clinical diagnosis, clinical trials, and research has been slow in part because of unclear cost advantages, but during the last 5 years, the technology has improved enough to play a larger role in diagnostic and research pathology. The recent Food and Drug Administration (FDA) regulatory clearance for five whole-slide imaging (WSI) systems for DP for primary diagnostic use in the United States (US) has been a major instrumental step forward.^1^ This clearance has and will help in widely and effectively adopting DP systems for clinical trials. Clinical trials are conducted in a regulatory environment with Good Clinical Practice (GCP) standards that ensure trial subjects' rights, safety, and well-being. Hence, all pathology-related activities in clinical trials also need to follow GCP standards.

The pathology parameters, including diagnosis, grading, staging, biomarker scoring, pathological complete response, resection margins, and recurrence, are key in clinical trials. Precision medicine advances have increased the use of IHC, in-situ hybridization, and molecular profiling for trial entry, patient stratification, and randomization. Clinical trial pathology differs from diagnostic pathology in that it uses masked or blinded assessments for randomization and clinical outcomes. Exploratory studies may use unblinded comparisons to identify treatment-related changes.^2^^,^^3^ In this review, we have tried to discuss the advantages, applications, challenges, and regulatory aspects of adopting DP techniques in clinical trials.

## Advantages of adopting digital pathology in clinical trials

DP notably enhances the efficiency of pathology reviews in clinical trials. It facilitates independent reviews by multiple pathologists and improves reproducibility between and within pathologists. DP also streamlines slide handling, enables centralized data management, and supports web-based consensus meetings. The salient advantages at each stage of a clinical trial are explained below:

### Central review, blinding studies, and digital peer review

In complex multicentric trials, samples are collected from multiple trial sites, which can be from multiple continents. Whereas testing at participating sites offers proximity to patient care, these complex multicenter trials often require a central lab with the necessary expertise and accreditation. A central lab in clinical trials is a specialized facility to manage sample collection, transportation, analysis, and data reporting applying stringent quality control. A central lab review is the preferred method adopted because it ensures uniform scoring criteria during patient pre-screening, which enhances the quality of the selection process for clinical trials. Central labs improve accuracy, reproducibility, and standardization. It also supports targeted therapy and precision medicine opportunities by providing a broader network of qualified pathologists with subspecialty expertise. Central pathology reviews are also designed as a quality control measure. Central review ensures uniformity and consistency of tissue processing through sample evaluations, by processing at a single lab with the same calibrated and validated instruments. Similarly, adoption of DP for central pathology review ensures standardized evaluation by trained pathologists, with easy access to remote second opinion and less logistical nightmare.

Although central labs have an advantage of control over analytical variables, they face challenges like sample logistics like shipment of glass slides, which can be overcome by DP that mitigates the risk of damage and loss. Central pathology review needs to be performed following data governance rules in a GCP environment.

DP adoption can assist in blinding and unblinding pathology evaluation and perform peer review with ease. In clinical trials, blinding studies with peer review is an important activity to avoid bias and maintain confidentiality. The adoption of DP is particularly beneficial here, as it facilitates a blind scoring workflow where cases can be distributed and reviewed by multiple pathologists while anonymizing patient data. This process generates robust, objective datasets for analysis. Blind scoring ensures that each pathologist independently evaluates and scores a case without being influenced by others, thereby enhancing diagnostic accuracy. Synoptic reporting, aided by DP reduces interobserver variability, which allows users to generate digital reports directly within the platform, improving the reproducibility of results across multiple reviewers and sites.

DP facilitates blinded peer reviews remotely via advanced image management software, which saves time and money. DP adoption enhances biomarker strategies and supports global clinical trials, which are conventionally challenging due to logistics. For instance, global clinical sites can process biopsies, ship them to local satellite labs for digitization which can be uploaded to cloud platforms and reviewed from anywhere on the planet. This allows for centralized pathology reviews without the logistical restrictions associated with physical histology components.^4^

### Training

Clinical trials involve multiple pathologists, in different centers, with diverse training and experience. The success of the trial depends on equitable training resources for all participating pathologists. DP allows flexible distance learning with a broader audience than traditional face-to-face training. Training may be required to standardize a feature that is routinely assessed in diagnostic work or to evaluate a new feature that is not routinely reported. This may necessitate a more comprehensive training program. A classic example is the training for clinical diagnostic pathologists to better understand the spectrum of HER2 IHC expression for identifying breast cancer patients who are eligible for treatment with Herceptin or ENHERTU. Similar training for pathologists working in clinical trials to interpret and score new IHC or IF biomarkers/companion diagnostic (CDx) tests can be easily implemented with DP. Pathologists participating from multiple central labs can be trained and evaluated efficiently for the review of these new biomarkers using DP. Developing new criteria in precision medicine, such as tumor-infiltrating lymphocyte (TIL) quantification or validated IHC quantification, necessitates digital learning methods for effective shaping of a clinical trial, underscoring the value of DP in training pathologists.^5^

### Gains in workflow productivity

A noticeable advantage of DP is the reduced need to physically move slides and tissue blocks, which mitigates the risk of damage or loss. DP adoption with central storage improves workflow by enabling flexible work schedules and remote access. Accessing archived digital slides is faster, which reduces search time and improves data matching and organization. DP speeds up sample utilization and improves processing time, especially in complex cases, and allows practices to be extended across geographic regions.^6^

### Digital archiving

Digital archiving offers a significant advantage by preserving digital images that can be accessed rapidly, contrasting with the time-consuming process of retrieving archived slides or folmalin fixed paraffin- embedded (FFPE) blocks for recutting. These archived images also serve as valuable training sets for new AI algorithms.^7^^,^^8^

When implementing digital archiving, considering the following key points is essential:

a) Organization and search efficiency: Digital slides are easily organized, enhancing the efficiency of searches.
b) Data volume: Whole-slide scanners can generate terabytes to petabytes of data annually. Regular clearing of caches ensures a seamless workflow.
c) Hybrid storage: A hybrid system, combining local hard drives with cloud storage, balances the cloud's extensive storage and collaboration capabilities with the rapid local access rates of a LAN.
d) Security: Cloud storage introduces the risk of data breaches if not adequately secured. Protecting sensitive patient information is critical. The Health Insurance Portability and Accountability Act in the US sets security guidelines for electronic protected health information, with comparable guidelines in many other countries.
e) Data encryption: Ensuring storage platform securely encrypts data both “at rest” on the local repository within a hybrid-cloud system and “in-flight” during transfer to cloud storage.
f) Staff training: Personnel must be trained in storing, assessing, and retrieving stored images on demand.

### Image analysis

Image analysis using artificial intelligence (AI)/machine learning (ML)/deep learning (DL), compared to subjective scoring, is one of the most important advantages of digitization, which cannot be performed on a conventional glass slide. Image analysis is highly relevant for clinical trials because it can objectively measure subtle differences between treated and placebo groups, thereby differentiating the treatment outcome. Additionally, although a general advantage of DP adoption, pathologists can view and compare multiple images in a single window, a feature not available in traditional glass slide review. This capability is particularly useful for identifying images/slides with subtle differences in scoring or severity.

The details of the image analysis process are discussed later in this review.

### AI and computational pathology

The field of computational pathology and the use of AI in DP depend on the digitization of glass slides. ML algorithms can automate the computational assessment of processes like evaluating TILs, scoring IHCs, tumor cellularity etc. which allows precise, rapid, and less-exhaustive workflows, improves diagnostic concordance, and adds information that cannot be assessed by the human eye. The details have been addressed in later sections. For example, creating an AI model that scores HER2, starts with large digital images of tissue samples, called WSIs. These large images are broken down into smaller, manageable, fixed-size pieces called “patches.” From these patches, an area is selected or also called “region of interest (ROI),” that most likely contains the cancerous tissue.^9^ Once the patches are ready, they are categorized into two groups, one for “learning” and one for “testing.” The learning group is used to teach the AI algorithm how to score HER2. The testing group is then used to see how well the algorithm is learned. This initial check, called “internal validation,” uses images from the same original set to measure the model's accuracy. To ensure that the AI's results are reliable and can be repeated, some models go through another step called “external validation.” This involves testing the AI algorithm's performance with a different set of images from a different source.^10^ Finally, the AI determines the overall HER2 score by adding up the individual scores from each patch it analyzed. Thus, because the whole process is more objective rather than being subjective, image analysis enhances the reproducibility of quantifying individual cells, leading to more robust data. An overview of salient details of computational approaches has been discussed in the section about exploratory biomarkers.

## Applications

The following section is an overview of some noteworthy oncological and non-oncological applications of using DP in clinical trials, which have eventually found a translational value. Although the list is not complete, it highlights how the adoption of DP has helped in clinical practice.

### Oncology

The role of digital and AI pathology in the field of oncology trials has various facets ranging from drug development, prognosis, and treatment response predictions. Due to the multifaceted nature of digital techniques, successful implementation requires a proposed multistakeholder group of experts, including but not limited to the central government regulatory committee, computational pathology software providers, trained AI pathologists and researchers, and biopharma industry key players, to outline a working model that can facilitate the correct implementation of computational pathology platforms for oncology trials and drug development.

The salient and most noteworthy practical applications of digital and AI techniques in the current times are:

a) Staging and prognostic predictions where one can achieve reduced throughput times and reader variability.
b) Quantitative measurements of TILs, which are proving to be an independent biomarker in today's era.
c) Measurement of IHC markers like ER, PR, Her2, and PDL1, which utilizes a semi-quantitative approach currently.
d) Cancer genomics applications, particularly, the prediction of MSI status, KRAS from HE slides.
e) Development of Ki67-like biological response marker in cancer treatment.

In the following paragraphs, we have tried to summarize examples in a few of the active areas where AI is helping in clinical trials.

The discovery and role of PDL1 and other targetable molecules in recent times have put AI pathology applications at the forefront, as the precise reporting of such markers and their location in the tumor microenvironment directly influences tumor therapy and response. As a result, the recent creation and approval of various software suites to measure these biomarkers have proven to be one of the core tools in any oncology-related clinical research.^11^

Ki67 IHC is commonly used as a prognostic marker in breast cancer. The international working Ki67 group (IWKG) recognized that the Ki67 IHC as a breast cancer prediction marker had clinical validity in selected cases of estrogen receptor-positive breast cancers. Ki67 analysis has had questionable validity, as the score is sometimes subjective, and a precise calculation is necessary, especially in the range of 5%–30%, where there is most subjectivity by human analysis. The IWKG tried to validate Ki67 scoring across three different image analysis platforms and found that the results had highly superior values statistically in terms of reproducibility. Thus, this underlines the use of DP in biomarker quantification, in a faster and reproducible manner, which ultimately benefits the patient as the data are dependable.^12^

Recent advent and trials in antibody drug conjugate drugs have led to further exploration of HER2 biomarker status in breast cancers, which now have an additional quantification criterion for HER2 low and HER2 ultralow categories. Recent studies, which have found that HER2-negative unresectable/metastatic BC may benefit from HER2-low-directed treatments, have successfully used digital and AI approaches for quantifying HER2 low status with reproducible and precise results, which otherwise would be time-consuming and subjected to subjective bias.^13^ For example, in a global, multicenter study, the challenge and the task were to ensure that pathologists from various labs could consistently and accurately rescore historical HER2-negative samples, with a specific focus on the finer distinctions required to identify HER2-low expression (IHC 1+ or IHC 2+/ISH−) and even finer subcategories like “IHC 0 with staining” (IHC > 0 < 1+). To address the subjective bias which can arise, especially to quantify membranous IHC such as HER2, pathologists were trained on a specialized proprietary software. The software provided a standardized self-study module where pathologists could review digital case collections of HER2-negative samples and receive immediate, automated feedback on their scoring performance. Following the training, the proficiency of the pathologist was tested using 30 test images. This digital format ensured that every pathologist worldwide was evaluated on the exact same set of cases, viewed under identical conditions, thereby eliminating variability that could arise from using different physical slides or microscopes. Thus, the digital approach used in the NCT04807595 study was not only AI-based but also centered on a sophisticated digital training and proficiency testing system. This further solidified that human pathologists were calibrated to a uniform standard, producing precise and reproducible data, essential for an effective clinical trial.^13^

DP and AI have recently been used efficiently in prostate cancer diagnosis and management. AI-based tools refine patient identification and selection for various purposes like prognostication and cancer detection in multiple samples over standard tools and allow researchers to computationally predict the likeliest outcomes in a precise manner, thus underlining their translational importance. Recently, the FDA approved, first AI tool for the detection in prostate cancers.^14^ AI tool deployment is being researched in multiple trials in prostate cancers.^15^, ^16^, ^17^, ^18^, ^19^, ^20^ The uses include:

1. Enhanced detection, targeted biopsies, grading, prognostication, and outcome prediction through imaging and pathology, with AI tools aiding diagnosis and treatment predictions.
2. Quantifying metastatic burden, identifying castration resistance, and predicting treatment responses, with multimodal data particularly in metastatic settings.
3. Monitoring and synthesizing new research for clinical guidelines and simulating clinical trials using generative AI, which can expedite research processes while reducing associated costs and time.

Microsatellite instability (MSI) is a pan-cancer biomarker approved by the U.S. FDA, allowing immunotherapy use in patients with MSI metastatic or unresectable solid tumors. Up to 15% of the colorectal cancers show MSI, and their detection plays a crucial role in the clinical management of colon cancers, with diagnostic, prognostic, and therapeutic implications. The conventional ways to evaluate this have been IHC, polymerase chain reaction, or next-generation sequencing. Recently, multiple studies have found the use of AI detection of this genomic alteration. One of the MSI detection tools called MSIntuit, described by Salliard et al. in colorectal cancer, recently obtained CE-in-vitro diagnostic (IVD) authorization and has been commercialized.^21^ The algorithm trained on The Cancer Genome Atlas (TCGA) dataset showed a high performance on an external validation on the AI platform.

Precision medicine approaches are advancing at a tremendous pace in this decade. And, the central approach (to select tumor samples and dissecting them down depending on the research question for patient enrollment or data production from pathology images) in making this possible is the use of different digital and AI pathology tools and methods. This includes but is not limited to H&E diagnosis to segregate cases, selecting ROI using specialized WSI softwares, which have plug and play tools like area selection, seeing multiple stains (sometimes even up to 60 markers) on a single slide, barcoding agents, which can help elucidate RNA and single cell topography of a tumor and nearest neighbor data of cell to cell proximities. A complete understanding of the tumor immune environment has important implications in immuno-oncology approach. The traditional approach has been to evaluate biomarkers using IHC with one marker at a time. Newer methods like multiplex immunofluorescence and spatial transcriptomics allow the study of tumor microenvironment very precisely using multiple markers at once and resolutions up to single cells. Different clinical trials search for different underlying problems, but central to every clinical trial utilizing these technologies involves a defined initial protocol generation for tumor immune microenvironment, generation of high-resolution digitized histology slides for ROI selection, and final analysis or elucidation of biomarkers (singly or co-expressed) by using an image analysis software and algorithm. These data can quantify as well as spatially resolve the data for meaningful results, which are translated from the bench to the bedside. Few notable studies and examples of these methods include multiplex signal amplification techniques (e.g., tyramide signal amplification), multiplex staining bleaching techniques (e.g., co-detection by indexing or CODEX), mass spectrometry imaging techniques (e.g., imaging mass cytometry, multiplex ion beam imaging), spatial high plex single cell technologies like NanoString GeoMx and Visium platform from 10× Genomics.^22^^,^^23^

The latest advancement in precision medicine has been to characterize organ microbiome,^24^ which is an intricate ecosystem with direct implications on metabolism, immune function, neuroendocrine activity, and tumor biology. The elucidation of human microbiome has been possible only because of AI techniques in pathology. Recent research using subjects from a phase I/II clinical trial [investigating atezolizumab (anti-PD-L1)] in combination with temozolomide and radiation therapy in newly diagnosed glioblastoma patients showed a role of microbiota in tumor biology.^25^^,^^26^ Similarly, another research showed gut microbiome is associated with increased immune cell infiltration in the tumor microenvironment and response to immunotherapy across cancer types.^27^ These studies used various spatial transcriptomic platforms and multiplex platforms, which were very central for meaningful data as shown by this research underscoring the advantages of DP platform adoption.

### Non-oncology applications

DP workflow platform is currently used in translational research and is being used for drug candidate screening and validation. This will also assist in uncovering biological mechanisms like underpinning NASH/MASH.^28^ DP with AI has been used for clinical trials in NASH studies, and this approach has shown greater sensitivity in demonstrating treatment-induced reversal of fibrosis in the liver.^29^ The combination of DP with AI is being used widely in clinical trials. DP with AI is a reliable and reproducible method that can both sensitively and continuously detect fibrotic changes in liver tissues of clinical trials.^30^

A recent phase II trial consisting of 251 samples evaluating AI scoring of liver biopsies in patients on semaglutide in non-alcoholic steatohepatitis (NASH) by Ratziu et al. using NASH ML models to quantify changes in different parameters using categorical assessments and continuous scores.^31^ Whereas the categorical scores generated by both pathologists as well as AI were comparable, the continuous score measured by AI was able to detect antifibrotic effect with superiority. Although the results do not imply a superiority of AI over pathologists, they certainly translate to refined details measured by AI which can sometimes escape the initial review, thus adding to meaningful results in a trial setting.

Several clinical trials in nephrology focusing on various aspects of kidney disease and treatment efficacy have utilized DP. Some of the notable trials include the evaluation of mucopolysaccharide inclusions in Fabry's disease in patients on chaperone enzyme replacement therapy,^32^^,^^33^ evaluation of proliferative lupus nephritis in patients using novel anti-TNF (Tumor necrosis factor) monoclonal antibody therapy,^34^ and quantifying the amount of transplant glomerulopathy after treatment.^35^ Various annotated WSIs in these trials have shown that there is less interobserver variability and better quantification of parameters, thus producing validated and precise results.

Whereas the applications of DP in clinical trials are broad, implementing these tools for specific endpoints like exploratory biomarkers requires careful management of technical and operational challenges, which are discussed in another section.

## Pros and cons of deploying digital pathology in clinical trials

DP implementation is a complex and integrative multidisciplinary process. A multidisciplinary team of IT department, lab personnel, pathologists, and management is involved. Whereas the strategic decision to implement DP is typically made at the level of management, it is essential to involve all units early in the process. The cost of DP implementation can vary significantly depending on several factors such as the scope of the operation, technology selection, location, and resources. Whereas there are significant upfront costs associated with the implementation of DP, the long-term benefits far outweigh the upfront infrastructure and training expenses.

### Initial hardware/software costs and maintenance

Acquiring the necessary hardware (scanners, updated computers/monitors, image management systems (IMS), and possibly servers) and software for DP can be a significant upfront expense. The cost varies based on the quality, capacity/throughput, and capabilities of the equipment and software chosen. Regular maintenance and updates are essential to keep DP systems running smoothly, which includes maintaining hardware, updating software, and ensuring data security.^36^^,^^37^ Recently, the Digital Pathology Association has developed an online calculator to assess and estimate the financial implications of moving to a DP system. This ROI calculator serves as a companion tool to assess potential costs and also savings due to DP systems.^38^

### Image integration

An efficient workflow is dependent on the interplay between different scanners and IMS. Most scanners can save images in different file formats or can convert them to different file formats, each with benefits and drawbacks. Commonly used formats include .tiff, .qptiff, .jpeg, .raw, .bif, .vms, .vmu .ndpi, and .jpeg2000. Lack of standardization leads to interoperability issues and thus can be a confounding variable in DP workflows in clinical trials, when different sets of slide cohorts are scanned in different scanners. The industry is moving towards adoption of the Digital Imaging and Communications in Medicine standard, which has long been established in radiology, leading to seamless interoperability and lower integration costs.^39^ Other potential solutions include the IMS to be able to integrate different image formats from different scanners, possibly with a tracking method, without affecting the quality of the image. Procurement of the equipment (scanners, up-to-date computers/monitors, image management platforms, and maybe servers) and software can be initially expensive. The cost depends on the quality, the capacity/throughput, and the capabilities of the equipment and software. Maintenance and updates are important to keep DP systems up and running, including hardware maintenance, software updates, and data security.^40^

### Color profiles and color calibration

Color management and color calibration of digital slides is very crucial for accurate diagnosis and effective patient care. However, color discrepancies in WSIs can result from various reasons, including but not limited to, specimen thickness, staining techniques, and the characteristics of the scanner used. This induces a very unique and challenging situation especially in multicenter clinical trials, where pathologists not familiar with the color profile or not aware of the color variation might lead to a bias, for e.g., in consensus evaluation.^41^ Effective color management involves understanding color reproduction and implementing regular color calibration protocols. Regular color calibration practices with standardized SOPs in a trial, using a combination of calibration slides and dedicated software can perform color management principles to correct images when faced with color and brightness inconsistencies, regardless of the microscope used to capture the images. This additional step might be limited too as various factors entailed earlier like specimen thickness, staining techniques can also induce minor irregularities in the calibration process, but is the best possible way to minimize errors.^42^ Finally, clinical grade medical displays are not feasible at each end user point, which can be a big confounding factor, e.g., in ascertaining nuclear details on a slide, validating the displays in such situations and keeping the user aware of such variations is a possible solution to minimize variability and bias.

### Digital storage of large volume whole-slide images

Storing large volumes of DP storage is expensive, especially for institutions or centers with large caseloads. Cloud-based solutions offer easy access to data but involve subscription costs. Data storage also becomes complicated with data safety, privacy, and regulatory framework around data storage in respective geographical regions. A major consideration factor for fast adoption of digital radiology is related to the size of the images and ability to retrieve them faster compared to the large DP images and retrieval time.^40^ Additionally, it is important to achieve an optimal and intuitive interface between the LIS and the IMS for easy adoption and work by the pathologists.

### Challenges in training

Training pathologists, lab technicians, and staff to effectively use DP systems is important. It is essential to recognize educational requirements and skills for efficient operationalization of DP infrastructure. Few practices may become obsolete over time, and it becomes imperative to retrain and revalidate the personnel regularly for not only expediting and continuing efficient implementation of the DP systems but also for regulatory compliance. Additionally, because clinical trials are a long commitment, retention of trained personnel is also crucial and sometimes a big challenge.

### Validation of digital pathology infrastructure

Integration and validation of DP infrastructure into existing lab workflows are complex, time-consuming, require training, and also incur additional costs. However, it is imperative to ensure compliance with regulatory requirements and standards set-up by the CAP (College of American Pathologists) and the FDA.^43^

### Opinion/learnability

Most pathologists still consider conventional microscopy as the gold-standard in terms of diagnostic confidence. As DP continues to grow, it is important to remember that most pathologists have been trained and have years, if not decades, of experience working with conventional light microscopes. The change in attitude and opinion will need soft persuasion and demonstration of tangible advantages. There has been a positive shift in the attitude towards DP, especially after the pandemic. Rapid turnaround times, easy and remote access with better ergonomics as compared to a conventional microscope are a definite advantage.

## Exploratory biomarkers and computational pathology in clinical trials: Overview and challenges

Exploratory biomarkers have become a key data component of clinical trials, enabling sponsors to generate evidence of therapeutic mechanism of action at the tissue level, which can be used to further supplement regulatory review and decision making on the main primary and secondary study read-outs. Exploratory biomarker data are also crucial in early-stage clinical trials, where timelines enable an understanding of pharmacodynamic (PD) read-outs that can measure disease state or predict patient response. These data can indicate confidence in success and dictate whether a sponsor will commit to more costly late-stage clinical trials.

### Value of utilizing digital image analysis (DIA) in generation of exploratory biomarker data

Quantitative DP is an established technique utilized to support the generation of exploratory biomarker data within early- and late-stage clinical trials during Pharma R&D.^44^^,^^45^ The combination of histochemistry, whole-slide scanning, and image analysis has enabled the generation of tissue- and cell-based quantitative data objectively from whole-tissue sections, providing datasets of superior depth and reduced variability compared with traditional manual scoring assessments.^46^ The utilization of these techniques has been further driven by the increased focus on immuno-oncology R&D over the past decade, with clinical biomarker programs requiring detailed quantification of immune-inflammatory cell populations, as well as their spatial localization, within the tumor microenvironment. This has been supplemented by advances in histochemical staining techniques including chromogenic and fluorescence IHC/ISH, multiplex, multimode (protein + RNA), and Virtual plex (same section H&E via image registration). In combination, this allows for the generation of whole-section datasets covering a wide range of read-outs, including marker area, cell counts, densities and co-expressed phenotypes, cell stain intensity (continuous, per cell), and spatial data *per* cell, *per* image.

The integration of AI, particularly DL neural networks, into image analysis algorithms has significantly advanced DP in pharmaceutical research. Unlike traditional ML methods that rely on manually engineered features—such as intensity, texture, or morphological descriptors—often derived from images pre-processed by techniques like color deconvolution and intensity thresholding,^47^ DL models automatically learn hierarchical features directly from raw images. This capability improves accuracy and generalizability across tissue types and staining protocols^48^ and has enabled the development of robust tissue and cellular segmentation tools critical for biomarker quantification and spatial profiling in clinical research.^49^^,^^50^ Ideally, algorithms will be generated during the translational R&D phase of the project, using images generated with the same histological staining and scanning approach to be utilized in the trial and tissue types consistent with the expected trial cohort. Development may involve a multidisciplinary group including image analysts, clinical anatomical pathologists, and data scientists, as well as input from the scientific project team. Although achieving algorithm generalizability across various tissue or cell types is desirable, due to the usually small cohort size of exploratory biomarker studies, and that read-outs will be unique to the clinical study in question, developed algorithms will generally not require broad use applicability across other tissues or staining applications, but will be focused on delivering high quality data from the specific clinical samples.

### Challenges of using digital image analysis for exploratory biomarkers in a clinical trial setting

Imaging biomarker exploratory endpoints in clinical settings require careful considerations about the entire DP platform and its level of compliance to ensure the images and data being generated are compliant for the intended use of the data. Recent advancements in biomarker assays and technologies such as multiplexing, imaging algorithms, and AI have significantly enhanced our ability to generate robust data. Imaging algorithms have become more sophisticated, enabling more accurate and efficient analysis of complex tissue images. AI further augments these capabilities by identifying patterns and making predictions that might be missed by human analysts. Despite these advancements, several challenges must be carefully considered to ensure the reliability, repeatability, and reproducibility and integrity of the data. A primary challenge is controlling the variability in biomarker staining and imaging of these samples to ensure consistent segmentation and detection of biomarkers in WSIs.

To achieve robust data, it is essential to standardize the DP workflow. This involves implementing rigorous protocols and quality control measures at every stage of the process, from sample preparation to image analysis. Ensuring that all steps are performed consistently helps to minimize variability and improve the reliability of the results. This includes regular testing and calibration of instruments, as well as updating algorithms to incorporate the latest advancements and address any identified issues. By maintaining a high standard of quality control and continuously improving the methodologies, researchers can ensure that the data generated are both reliable and actionable.^51^^,^^52^

#### Biomarker variability

Biomarker staining variability poses a significant challenge to reliable cell and tissue segmentation, particularly when studies are conducted across multiple labs. This variability can lead to inconsistencies in the data, making it difficult to draw accurate conclusions. To address this issue, it is essential to implement continuous testing and measurement of several key factors during biomarker optimization.^53^

Biomarker assay variability must be rigorously monitored. Assay optimization of these biomarkers to generate the optimal signal-to-noise ratio can be challenging and should be monitored by a pathologist. Optimization should incorporate automated staining platforms using commercial reagents that undergo rigorous quality checks to ensure consistent results.^54^^,^^55^ Once an assay is approved, the protocol should be locked and intra- and inter-variability studies should be carried out across different instruments, sample types, personnel, and sites to establish performance and concordance baselines. This is important to monitor new lots of reagents, personnel, instruments, and sites.

#### Imaging variability

Imaging variance is a critical aspect of standardization in DP. Even if biomarkers are optimized for their staining, the actual scanning or digitization of WSIs can be significantly influenced by the instruments used, which vary by vendor. This variability can impact the consistency and reliability of imaging data.

In clinical settings, it is essential that scanning instruments, IMS, and viewing software are FDA-approved to ensure they meet stringent quality and safety standards. The CAP has developed guidelines to validate entire imaging platforms for Good Laboratory Practice by following GxP guidelines.^56^ These guidelines provide a structured approach to ensure that imaging systems are reliable and produce consistent results. For non-GxP studies, researchers should also incorporate some level of validation standard operating procedures (SOPs) into their workflows. This integration helps to maintain consistency in scanning, image management, and viewing processes, even when the studies do not fall under regulatory requirements. By doing so, researchers can ensure that their imaging data are robust and reproducible.

Brightfield scanners creating WSIs are the first step in creating high-quality images, without consistency, these images face significant challenges when performing imaging. These include issues related to tissue quality, such as poorly sectioned samples, uncalibrated instruments that can produce lines or out-of-focus areas in the images. Additionally, dirty slides and artifacts from cover slipping, such as bubbles, can further compromise image quality affecting downstream imaging outputs.

Fluorescent scanners, which are used to image tissues labeled with fluorescent biomarkers, present their own set of challenges. Variability in the exposure of different biomarkers across a tissue section can lead to significant differences in image quality. Areas with artifacts or regions where biomarkers are weakly or intensely expressed can make it difficult to achieve accurate exposures across the entire tissue. This variability can complicate the analysis and interpretation of the imaging data.

Image management can also pose several challenges that affect the ability to store and view WSIs and metadata. Proprietary image file formats with specialized viewing software make it difficult to manage imaging data from one imaging platform to another. Image storage can pose a major challenge to imaging studies, from images being generated across external and internal labs and a major commitment to building and supporting by internal IT departments and management. There are a number of DP platforms that offer options for storing and viewing images and associated metadata which can accelerate managing images, but it is important to understand the cost and capabilities of these platforms and the significant resources and stakeholder acceptance.

Therefore, by implementing these measures, researchers can enhance the consistency and reliability of their biomarker staining and imaging processes. This, in turn, will lead to more accurate and reproducible data, ultimately improving the quality of scientific research and its applications in clinical settings.

### Increasingly complex staining assays

Multiplex assays involve the simultaneous detection of multiple biomarkers add another layer of complexity. These assays require thorough validation to ensure that each biomarker performs consistently, whether it is used alone or in combination with others. This validation process should include testing for cross-reactivity and interference between biomarkers, as well as ensuring that the signal-to-noise ratio is optimized for each marker and the performance is the same as a singlet or in a multiplex assay.

The development of spatial biology has driven the development of highly plexed biomarker assays with technologies capable of approaching Hi-content multiplexing of biomarkers in tissues. Understanding the ability to multiplex these biomarkers requires a deep understanding of how these biomarkers interact requiring knowledge about low vs high expressors and assumptions around acceptable predictive steric hindrance when layered with subsequent biomarkers.

There are a small number of biomarkers currently validated for clinical use, such as PDL1, ER, PR, and HER2, which are usually CDx assays that accompany a specific drug to diagnose target expression to identify patients for entry into a trial and monitor the drug's efficacy post treatment. The majority of these biomarker assays are singlet, but multiplex assays’ use as exploratory endpoints in clinical trials is increasing.

Given that there is no standardized assay capable of detecting all antibodies, the technology employed to conduct the assay will dictate the approach to validation for the inclusion of a biomarker in a multiplex assay. Generally, antigen retrieval is important to consider because cycling will expose these biomarkers to different retrievals during the staining process. It is important to look at different antigen retrievals during the initial optimization of any biomarker to understand its performance and guide its placement in the cycle.

For discovery research, Hi-content multiplex can generate valuable data to identify potential targets, however, the sheer number of biomarkers regardless of the technology require significant assumptions that there is no drop in performance of each biomarker. For clinical samples, the number of targets or multiplex needs to be carefully considered from a separation standpoint during the imaging whether there is sequential imaging or static imaging at the end of the assay. This is important when developing a clinical assay that requires stringent validation, so the lower the number of biomarkers is more manageable.

In summary, managing multiplexing exploratory biomarkers in clinical samples requires number of parameters that need to be considered and addressed to ensure the integrity and performance of each biomarker to generate actionable data that can be used to identify trends in a drug's efficacy in clinical trials.

## Regulatory considerations for digital pathology in clinical trials

The deployment of DP tools within a clinical trial requires careful preparation by sponsors and investigators to ensure compliance with requirements and successful use of results. However, a standard playbook on how to achieve compliance when deploying these tools in clinical research does not exist. Therefore, sponsors and investigators are often faced with weaving together a patchwork of regulations and standards that are not always directly topical or prescriptive into compliant quality processes and protocols. To assist with undertaking this challenge, we have provided recommendations from DP experts on the primary regulations and standards to consider to compliantly utilize, validate, and deploy lab tests with electronic applications in a clinical trial.

**Fig. 1:**
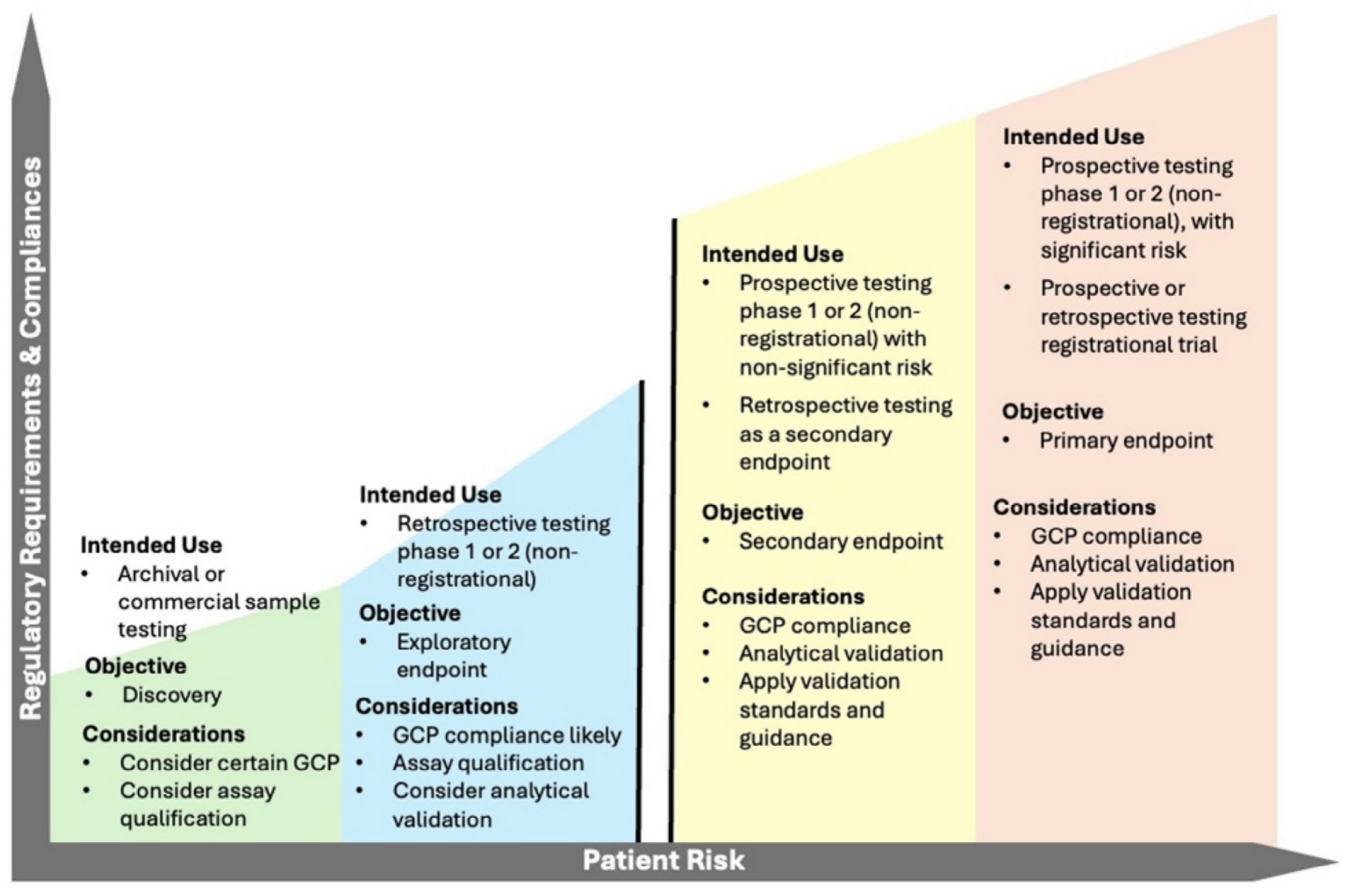
A clinical trial study design determines this risk to the patient who participates in the study. The greater the risk to the patient, the greater the requirements that must be met, regardless of the phase of the clinical trial. The intended use of a digital application or tool in a clinical trial is the largest factor that drives the regulatory requirements. Prospective testing, in which a lab test result supports eligibility for enrollment in the clinical trial, carries greater risk than retrospective testing, in which the result does not have an impact on patient management. The use of the result to meet a study objective (i.e., endpoint) also impacts the level of compliance, which can be a separate but not mutually exclusive consideration. However, the permutations of a clinical study design can be endless, and each clinical study must be considered independently.

The various aspects of a study design, whether the patient population, use of the test results, sample collection method, test methodology, or other details, create nuances in how to approach compliance. The intended use of a digital application or tool in a clinical trial is the largest factor that drives the regulatory requirements for its use, documentation, validation, and health authority approvals. Results that support a primary or secondary endpoint only relevant within a clinical trial, such as measurement for a PD endpoint, have a different context than an application intended to be commercialized as IVD, such as a CDx, and are associated with different regulations. Using the latter as an example, the use of a test result (e.g., biomarker positive or negative) to define eligibility for a clinical trial, often known as prospective testing, can require the highest level of compliance with regulatory requirements. The requirements are high because the results affect the medical management of a patient, which increases the risk to the patient. As a general rule, consider the greater the risk to the patient, the greater the requirements that must be met (Fig. 1). This statement is true regardless of the phase of the clinical trial. For example, if a biomarker hypothesis is being tested in a phase 1 clinical trial, but prospective patient selection for eligibility is being applied, regulatory requirements remain high.

Whether phase 1 or phase 3 of the clinical trial, the use of a test result in a clinical trial often minimally requires compliance with GCP standards. The sponsor then further deconstructs the implications of the intended use, additional expectations for component and/or system validation, clinical lab standards, health authority authorizations, and potentially medical device standards must be considered (reviewed further in Section on Compliance requirements for Investigational IVD Use in Clinical Trials). As an example, the impact of lab results on study objectives also affects the required quality standards and validation. An increased impact, such as for a primary endpoint, requires more analytical validation evidence and regulatory compliance to ensure data integrity and the generation of robust evidence for safety and effectiveness (reviewed further in Section on Strategies for ensuring compliance).

### Guidelines for compliance regarding the use of investigational IVDs in clinical trials

When considering certain general use cases, a blueprint of compliance with regulations and standards can emerge. Here, we summarize a few of the major compliance considerations for the US and European Union (EU), and a deeper dive can be found in the Appendix. Unfortunately, however, the permutations of a clinical study design are endless and can make it impossible to create a playbook to use for each permutation. Therefore, the recommendations in this review should only be taken as a potential starting place. It is important to utilize Regulatory Affairs and Quality Assurance professionals to guide the compliance approach for an individual trial.

In the USA, the use of an investigational application within a clinical trial requires consideration of Investigational Device Exemption (IDE) regulations under Title 21, Part 812 of the Code of Federal Regulations (21 CFR 812).^57^ These regulations must be considered even when the testing may occur in a CAP-accredited or Clinical Laboratory Improvement Amendments (CLIA)-certified lab. It is important to recognize that the FDA oversees the IDE regulations, whereas the Centers for Medicare and Medicaid oversees clinical lab regulations, like CLIA. Importantly, this oversight is separate and must be considered independently. An IDE is a regulatory submission that must be approved by FDA (specifically, the Center for Devices and Radiological Health) before the use of a DP investigational IVD in a clinical trial when the study design poses a significant risk to a clinical trial participant. A sponsor is responsible for initially determining if the study design represents a significant risk to enrolled clinical trial participants, and an independent review board and/or FDA will then make a decision on the sponsor's assessment through a “study risk determination.”

Although the clinical lab regulations are separate from FDA oversight, certain clinical trial designs will require compliance with CLIA (42 CFR 493), such as if the use of an assay affects patient management (e.g., prospective enrollment). In this context, CLIA must be adhered to in addition to FDA regulations. Therefore, when the use of results affects patient management in a clinical trial, oversight by two regulatory authorities applies, but do not overlap.

When considering a global approach, it is important to assess the similarities and principal differences in regulatory requirements between global regions. Using the US and EU as comparative examples (Table 1), both regions require human subject protections and an independent review of a protocol to initiate clinical research in any phase. These concepts are defined in GCP standards, which are an internationally recognized set of ethical and scientific principles intended to assure that the rights, safety, and well-being of clinical trial subjects are protected and that the clinical trial data are credible. However, the additional regulatory requirements can differ between the regions based on the study design.

**Table 1:** Salient regulatory similarities and differences between the US and EU regions.

	United States	European Union
General requirements	Requires human subject protections and independent review of a protocol to initiate clinical research in any phase (US Code of Federal Regulations)	Requires human subject protections and independent review of a protocol to initiate clinical research in any phase (Member State Local Laws)
Governing standards	Follows GCP standards	Follows GCP standards
Investigational IVD use	The use of an investigational IVD in a clinical trial requires consideration of IDE regulations under 21 CFR 812	The use of an investigational IVD under the IVDR requires consideration of Articles 57 & 58, as well as Annexes XIII & XIV
Risk-based approach	FDA approval of an IDE is required if a study design poses a significant risk to a clinical trial participant.	Increased risk to the patient, such as an interventional trial and/or invasive sample collection, requires performance to meet additional requirements, such in Annex XIV. Annex XIV requires NCA authorization to begin the trial.
Study risk determination	A sponsor determines study design risk. An IRB and/or FDA will then make a decision on the sponsor's assessment through a “study risk determination.”	If an assay has a medical purpose, a sponsor must then determine the applicable IVDR. Article 57 defines the requirements for any device for a performance study, and Article 58 outlines the requirements for increased risk.
Patient management	If the use of an assay affects patient management (e.g., prospective enrollment), clinical lab regulations, specifically CLIA (42 CFR 493) must be met in addition to FDA regulations.	If an assay has a medical purpose within the clinical trial, then the sponsor must determine the applicable requirements under the IVDR.
Electronic tools	The use of electronic tools requires compliance with 21 CFR 11 and applicable GCP guidelines	The IVDR has requirements for electronic systems, and conformity with GCP is expected.
Compliance standards	Sponsors can achieve compliance for computer systems in a setting in which GCP would be applicable, through recognized standards, like GAMP 5.	Standards like GAMP5 could be used as a framework for international compliance

The US has been discussed above, and in the EU, the implementation of the In Vitro Diagnostic Regulation (IVDR) in May 2022 significantly increased the requirements for the use of an IVD in a clinical trial, which includes DP devices.^58^ Under the IVDR, if an assay or tool, such as a WSI system, has not obtained a CE mark, specifically for the intended use within a clinical trial, it must be determined if the assay is defined as a device for a performance study and requires compliance with relevant articles/annexes of the IVDR. To determine if the IVDR applies (i.e., it is a device for a performance study), the sponsor must determine if the use of an assay has a medical purpose within the clinical trial, which has been defined in guidance published by the Medical Device Coordination Group.^59^ If an assay has a medical purpose, a sponsor must then determine the applicable requirements that must be met under the IVDR.

Article 57 defines the requirements for any device for a performance study, which includes compliance with general safety and performance requirements (GSPRs). Compliance with GSPRs can carry robust requirements for the manufacture and validation of the assay before its use, significantly increasing the effort for use of a DP investigational device in a clinical trial. It is important to note these requirements apply even in early phase clinical trials even when the use might represent hypothesis testing.^60^

Much like the US, risk to the patient also contributes to the regulatory requirements that must be met. For example, an interventional trial and/or invasive sample collection represents increased risk and Article 58 requirements, including those set out by Annex XIV, must be met. In most instances, Annex XIV requires the submission and authorization of an application by the Member State National Competent Authority for each country in which tests will be reported. Submission to an Ethics Committee (EC) must also be considered per local country laws.^61^

Because DP systems are electronic systems, further regulations and standards must be considered across global regions. In the US, 21 CFR 11 defines the criteria under which the integrity and security under which electronic records must be maintained. GCP also outlines the need for validation and integrity in the use of electronic systems. However, 21 CFR 11 and GCP guidelines are not prescriptive on how to achieve compliance. Therefore, sponsors often utilize standards that provide greater detail on how to achieve compliance for computer systems, such as the standard published by the International Society for Pharmaceutical Engineering (ISPE), GAMP 5. This is an internationally recognized standard, and so, could be applied in a global context. ISPE also recently published an article that provides a recommended approach to implementing guardrails that ensure the quality, privacy, and security used in AI applications. Standards published by ISPE could be used as a framework for international compliance.^62^

### How to achieve compliance for data generation in clinical trials

**Fig. 2:**
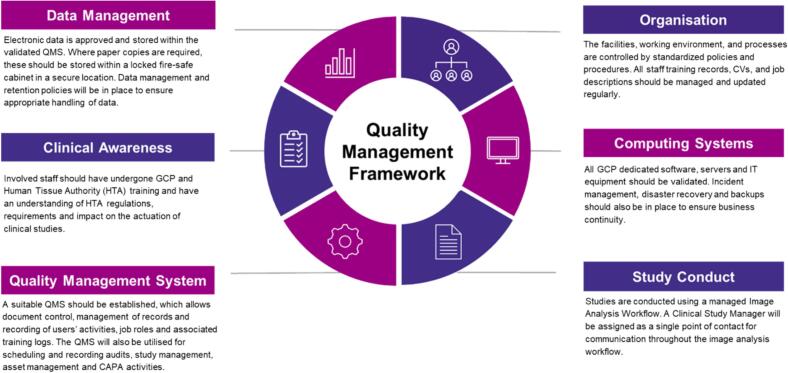
An outline of an example quality management framework.

In line with GCP guidelines, any lab performing DP activities to a regulatory standard is expected to have in place processes and systems able to support and manage quality during the clinical trial period. To this end, the establishment of a quality management framework (QMF) that aligns with a quantitative image analysis workflow suitable to support studies performed to GCP should be created (Fig. 2). The QMF should address several aspects within the DP environment required to demonstrate and support the delivery of robust and high-quality data within a workflow that is both transparent and auditable. This may include details of the company organization, computing systems, study conduct process, data management, clinical awareness, and outline their quality management system (QMS).

At the heart of the QMF will be a QMS set-up to manage all aspects of the clinical trial data requirements, particular to that company or CRO. The QMS can be a repository for documentation such as SOPs, policies, and templates, together with management of suppliers, assets, audits, and also corrective and preventive actions, integral to supporting quality assurance (QA) guidelines to address any potential or confirmed non-compliance events, either with the GCP protocol or processes involved. Many commercial QMS software are available and are selected based on specific company requirements.

Appropriate training of staff involved in GCP image analysis studies, along with an assigned QA independent of a study, is required to ensure a focus on quality and compliance. An agreed and transparent workflow should be detailed in the study plan that requires clinical pathologists to work closely with image analysis scientists to support tissue annotations, algorithm development and validation, ensuring the most reliable data generation. The algorithms developed for the image analysis of clinical tissue images to a regulatory standard are required to undergo validation. This serves to compare the accuracy of an algorithm’s automated classification or quantification read-outs against a clinical pathologists' ground-truth annotations and should be detailed as part of the image analysis study plan. Following rigorous review and approval through a series of defined and auditable steps, the validated algorithms can be applied to study images. All data generated should be quality control checked and aligned with the QA assessment of the processes required to produce that data, as part of an auditable workflow. Successful conclusion of this study workflow requires effective communication between the sponsor and the services lab through continuous review and milestone decisions in the study.

### Future outlook and needs

The inclusion of DP applications within clinical trials is augmenting data packages, aiding regulatory authority decision-making and positively impacting progression of therapies to market. Fundamental to this process is the compliance guidelines and standards required to ensure a focus on quality and safety. Regulatory guidance currently provides clarity for studies utilizing DP in generation of primary data and IVDR use. However, it is less so for secondary data and also in the instance where retrospective exploratory data have identified a biomarker signature, for example, between samples from trial responder and non-responder subjects, which may be used as the basis for future therapeutic CDx development. Further clarity on regulatory requirements across these scenarios will help ensure that trial design, preparation, and execution are appropriately in place to benefit from the increasing value provided by DP.

## Future developments and considerations

### Integrated diagnostics

Clinical trials are multidisciplinary and require a wholesome approach to dissect the research question and ultimately be scalable to the user market. The gold-standard for diagnosing and understanding the morphology of diseases is histopathological analysis. The advancements and breakthroughs in DP have enabled the field to progress into the realm of computational pathology, which if integrated with other imaging modalities and -omics can lead to the “precision” in precision medicine and hence successful and meaningful clinical trials.

Pathology, radiology, and genomics are siloed diagnostic disciplines. Data across these modalities can help to create better CDx tools. Pathomics or computational pathology helps to extract minable data from the morphometric measurements or IHC quantitation. Similarly, radiomics refers to quantitative measurements of textures and shape attributes, extracted using high-throughput image processing and computer vision from radiological images. Genomics focuses on gene expression data to gain molecular insights, by investigating mutations, mutations, copy number changes, DNA methylation, and gene expression. Regardless of the modality, the data outputs can be merged with the help of computational capacity and an integrated report can be created to deliver actionable insights in this era of precision medicine.

For example, a recent study by Vaidya et al. focused on combining radiomics using CT scans and pathomics from H&E slides to create superior predictive models for three critical clinical scenarios in lung cancer management, which were, predicting disease recurrence in early-stage non-small cell lung cancer (NSCLC), forecasting immunotherapy response in advanced-stage NSCLC, and assessing chemotherapy response in small cell lung cancer (SCLC). The integrated ES-MRP model (Early Stage–Radiomics Pathomics) significantly outperformed the individual radiomics (ES-MR) and pathomics (ES-MP) models in predicting disease recurrence. More specifically, textural features from the tumor and surrounding area (radiomics), and spatial features (characterizing the spatial arrangement of TIL and cancer cells) (pathomics), when combined, performed superiorly as compared to either of the technologies. Similarly, in the same study, combining nuclei shape on HE slides morphology to Haralick entropy (potentially indicating hypoxic regions) and peritumoral Gabor features (associated with inflammatory cells), which were extracted on the radiology images, were more superior to predict immune checkpoint inhibitor therapy response in patients.^63^

Digital and computational pathology solutions, in a way, can learn from genomic platforms that matured over the last three decades, and have become an integral part of clinical trials. Genomics has firmly established itself as a cornerstone in the design and execution of clinical trials, particularly in precision medicine. Genomic data often serve as a primary selection criterion for patient enrollment, enabling the identification of subpopulations that are most likely to respond to a given therapy. For instance, the presence of specific mutations, e.g., the presence of EGFR or KRAS mutations, can dictate the inclusion of patients in trials for targeted therapies.^64^ Beyond patient selection, tumor genomics frequently contributes to secondary analyses, helping to elucidate mechanisms of resistance, identify biomarkers for efficacy, and stratify patients by risk.

The integration of genomics in clinical trials is not only enhancing patient selection but also accelerating the development timelines by providing more precise data early in the trial process. This approach can lead to earlier go/no-go decisions, reducing the time to market for new therapies. The changing landscape of CDx, which increasingly incorporates genomic data, reflects this shift towards more integrated and precise approaches in drug development. Genomics allows for dynamic monitoring of disease progression or treatment response through multiple time-point read-outs. For example, serial liquid biopsies can be used to track the emergence of resistance mutations in real-time, offering the possibility to adjust therapeutic strategies before clinical resistance becomes apparent. In trials, this approach can be crucial for adapting treatment protocols based on evolving genomic profiles, thereby improving outcomes and optimizing trial efficiency. In other words, genomics has accomplished the firm integration by demonstrating added value in many (if not all) areas of drug development.

Integrating genomics with anatomic pathology and radiology enhances the ability to develop comprehensive, multidimensional diagnostic insights.^65^^,^^66^ For example, a study by Braman et al., explored the use of integrating the three modalities, i.e., radiomics, pathomics, and genomics in glioma patients. The approach involved generating *embeddings* (learned feature representations) for each modality, which were then combined using an advanced technique called attention-gated tensor fusion, a crucial step as it captures all possible interactions between the different data types (e.g., combining the radiology features with the genomic features). To ensure complementary nature of the different modality data and that each data type contributed uniquely and meaningfully to the model, they applied a multimodal orthogonalization loss. This mathematical constraint helped maximize the information derived from each modality, ensuring they were truly complementary rather than redundant. In summary, this integrated, multimodal prognostic signature significantly outperformed the best performing model that relied on only a single modality (unimodal model).^67^

In a recent presentation at the Pathology Visions conference 2025, it was showcased that the use of AI-driven computational pathology can predict the likelihood of the AKT1 E17K mutation, a clinically actionable variant in lung adenocarcinomas associated with response to specific targeted inhibitors, demonstrating potential to infer genomic and molecular features directly from H&E-stained histopathology slides.^68^ The digitized H&E-stained slides were analyzed using AI algorithms to assign a mutation probability score and patients labeled as AKT1 positive could be prioritized for confirmatory molecular testing. This algorithm is based on previous work which showed that driver mutations in lung adenocarcinoma can be rapidly detected from whole-slide H&E-stained images.^69^ The entire workflow from uploading a scanned HE slide to a cloud-based platform and analysis makes a streamlined and scalable system for patient stratification using DP. The cost of a confirmatory molecular testing (NGS) is approximately 20 times higher than that of the computational pathology-based screening, underscoring the potential for substantial cost- and time savings. The integration of computational pathology into clinical trial workflows therefore offers a promising strategy to enhance the efficiency of patient stratification, reduce operational costs, and accelerate recruitment of molecularly defined cohorts.

### Virtual staining

Despite advances in downstream DP image analysis, including AI-based decision-support and pre-screening, a major bottleneck remains the initial generation of images. Even at the largest institutions, most traditional DP deployments have duplicated technical efforts, requiring both the typical physical generation of glass slides and chemical staining and then subsequent scanning, thereby effectively extending the lab workflow. This workflow would be analogous to using traditional film for print-based photography, developing hard-copies, and then scanning those copies for the purpose of obtaining digitized images for digital manipulation and alteration. In other words, until recently, DP lacked the ability of consumer digital cameras to bypass the physical medium of chemical staining and generate purely digital, chemistry-free images. Recently, the development of the field of virtual/computational slide staining (VSS) has upended that paradigm by transforming a 150+-year-old histopathology workflow.^70^

The first generation of VSS used non-brightfield imaging methods to generate specimen contrast with techniques such as Raman, hyperspectral imaging, and photoacoustic microscopy, among others. As pathologists are not trained with these types of contrasting methods, attempts were made to transform these into pseudo-H&E images, by mathematically calculating approximated dye absorption. However, the resulting images lacked the structural, cellular, and intracellular compartment detail of a high-quality H&E, and they also appeared unfamiliar and foreign to pathologists, typically providing only lower magnification, triage-level review. Furthermore, these were incompatible with safety and toxicology workflows in which focal changes—requiring high magnification review—and precise color fidelity are mandatory to identify subtle changes. As a result, these technologies remained relevant for only niche applications.^71^

More recently, breakthroughs in the field of ML and deep neural networks have allowed the production of high fidelity, diagnostic-grade VSS. One such technique, pioneered by and optimized by a VSS company, uses a deep neural network training using supervised learning with accurately co-registered patches of paired images to convert native autofluorescence signals on label-free/unstained tissue sections into VSS. The images are acquired using available fluorescence microscopes. This transformational technique generates H&E images nearly indistinguishable from their chemical counterparts as well as a host of virtual special, immunohistochemical, and immunofluorescence stains that are immediately pathologist viewable with no specialized training.^72^

The workflow and life-cycle benefits of such a deployment are legion: force-multiplying the output of a dwindling histotechnologist workforce by allowing a single tech to perform more tasks; bypassing existing operational choke points; standardizing stain quality through reduction of batch to batch variability with consistent staining, regardless of the specimen's physical location; allowing a pathologist viewing a suspicious H&E to request an instant reflex to an ancillary supportive stain that could be generated within minutes and that will perfectly aligned with the original stain; reducing the usage of input chemicals, reagents, and water usage, thereby reducing cost, improving supply chain resiliency, and minimizing toxic exposures to histology lab staff.^73^

By leveraging AI algorithms, the histology workflow can thus be compressed, waste generation can be reduced, turnaround time can be improved, and oncologists can get more rapid answers to expediently place patients on definitive treatment. Furthermore, non-destructive staining can be performed on blocks scanned at an earlier time point, allowing unstained slides scanned at an earlier phase of a trial to be subsequently interrogated months or years later with no stability issues that often plague cut sections. Such capabilities not only allow exploration of innumerable biomarkers on diminutive specimens but also preserve tissue in the event of regulatory requirements for bridging studies, which often plague CDx development when transitioning from an earlier LDT-CTA to an IVD-CDx. Whereas the novelty of this technology renders it most effective in translational, discovery, and exploratory analyses, its potential for future clinical deployment remains untapped.^74^

### Example of use of newer modalities for companion diagnostics (CDx)

The black-box nature of certain newer computational pathology modalities poses challenges for patient selection programs which may culminate in a class III CDx designation. At the recent 2024 World Conference on Lung Cancer (WCLC), AstraZeneca announced a novel computational modality based on a quantitative continuous score normalized against the membrane to cytoplasmic ratio (QCS-NMR) for an in-development TROP2 ADC.^75^ This method started from a visually recognizable, standard chromogenic IHC stain for TROP2 but created a novel interpretation method to calculate the differential expression of optical density of the membrane vs cytoplasm of TROP2 positive cells. Such a method, while conceptually understandable, would be completely unreproducible for human pathologists but introduces the possibility of predicting responders vs non-responders using metrics and signals only detectable by ML algorithms. The immediate interest and vigorous debate over QCS-NMR at WCLC confirmed that the industry is ripe for such algorithms to be deployed, but significant work remains to be completed as such algorithms cannot be plausibly considered robust if datasets used to train the model largely overlap with datasets used to test the model. Furthermore, such models currently appear to only have been tested in a retrospective fashion as an exploratory endpoint which was not pre-specified, raising the possibility that such correlation may not be borne out in future prospective, pre-specified studies. Additionally, the degree of generalizability of such models which incorporate both a classic wet lab technical component and a dry lab analysis/AI component remains to be seen. Given that ML algorithms are exquisitely sensitive to changes in ground truth, slight changes in outputs from the wet lab component (e.g., intensity of IHC staining) combined with slight changes in calibration characteristics of imaging components (e.g., color palette and gain of slide scanning equipment) can result in drastically different results output from the AI model. For a CDx program, all of these parameters will need to be robustly locked down, analytically verified, and clinically validated, a process which currently takes years for a classic chemistry-based assay and could easily be extended further out if AI were to be added. Regardless, given the public relations success of early attempts at computational CDx programs such as QCS-NMR, we believe there will be sufficient market drive and incentive for stakeholders to fully explore these possibilities in collaboration with regulators.

### Foundational AI models

A limitation of all AI-based technologies, including virtual staining and user-assistive decision-support tools, is the broadness of the use case within which they can be applied. Many AI-based technologies can only be applied in relatively narrow use cases. For example, a decision-support tool to draw attention to metastatic carcinoma in a lymph node may not have been trained on variant histologies, sarcomas, or lymphoproliferative disorders and may fail to accurately detect these; alternatively, an AI-based tool to virtually H&E stain liver tissue may not have been trained with various non-hepatic origin metastatic diseases that can appear within the liver. The two primary approaches to expanding the usability of such technologies involve layering on multiple overlapping AI feature sets—for example, training multiple AI algorithms and applying them sequentially or simultaneously within a dataset—or alternatively training a very broadly encompassing or so-called “foundational” model on an enormous dataset, which is expected to perform accurately in a broad range of environments. These foundational models can often require tens of thousands to hundreds of thousands of datapoints and slides, encompassing nearly all frequently and infrequently encountered normal, inflammatory, neoplastic, and toxicological histologies as well as variant pre-analytic and analytic parameters such as fixative type, fixation time, slide thickness, target retrieval solutions, stain characteristics, scanner types, and myriad other parameters, typically assessed from multi-institutional cohorts. Whereas foundational models provide the broadest usability and confidence for end-users, the staggering data requirements for training such models have mostly precluded their development, though various consortia in the private and public sectors have been advancing their usage as computational pathology grows more relevant for precision medicine.^76^, ^77^, ^78^ Thus far, the majority of such foundational models have focused on identification of diagnoses and diagnostic parameters, although upstream technologies such as foundational virtual stains are also being explored to enable computational staining across pan-species tissue samples.

## Conclusion

Clinical trials are a very complex activity performed in a vast multidisciplinary setting.

The technology stack in clinical trials is rapidly evolving through advanced digital transformation. Generative AI, ML, AI powered automation, agentic AI etc. are streamlining protocol authoring, regulatory document preparation, patient recruitment, and data analysis, which are reshaping clinical trials making trials faster, more efficient, and increasingly patient-centric. Integration of radiology reporting in clinical trials using digital platforms has already advanced in its digital transformation. Pathology is one of the only components of clinical trials which has been slow in digital transformation. Pathology is the key to interpret the drug target interactions in clinical trials for identifying the efficacy and safety of a drug. Accelerating the pathology digitization process provides the integration of histopathological and molecular features with spatial context along with adoption of AI/ML/DL will enable more accurate patient stratification, optimizing precision clinical trial design, leading to accurate interpretation of trials data. Through this review, we have tried to address most important issues and possible scenarios of usage and implementation of DP in a clinical trial setting and hope that in addition to our literature and other future literary works, refining takes place in the future to seamlessly integrate DP in clinical trials.

## Declaration of competing interest

The authors declare the following financial interests/personal relationships, which may be considered as potential competing interests:

**Table:** 

Harsh Batra reports article publishing charges were provided by the Digital Pathology Association. If there are other authors, they declare that they have no known competing financial interests or personal relationships that could have appeared to influence the work reported in this article.

## Appendix A Supplementary data

Supplementary material Supplementary data (Regulatory standards and guidelines to consider for digital pathology in clinical trials). Supplementary material
