# Supplementary material for: Applications and challenges of utilizing digital pathology and AI-enabled workflows in clinical trials
Source: J Pathol Inform. 2026 Jan 2;20:100542. doi: 10.1016/j.jpi.2025.100542 (PMC12861029; doi:10.1016/j.jpi.2025.100542)
Supplement: Supplementary material — Supplementary data (Regulatory standards and guidelines to consider for digital pathology in clinical trials). [file mmc1.docx]

**Supplementary**

Table 1. Select Regulations, Standards, and guidelines to consider for digital pathology in clinical trials

| **Document Number** | **Title** | **Description** | **Recommended Compliance** |
| --- | --- | --- | --- |
| 21 CFR 11[1] | Electronic records; electronic signatures | US regulation that defines the criteria under which electronic records, electronic signatures, and handwritten signatures executed to electronic records to be trustworthy and reliable. | Compliance is needed when an electronic system or tool is used to support activities for a clinical study. |
| 21 CFR 50[2] | Protection of Human Subjects | US regulation intended to protect the rights and safety of subjects involved in investigations files with the Food and Drug Administration. | Compliance with this regulation is thought of broadly and extends to samples collected from a human to be used in clinical research. Informed consent for the use of samples is necessary either as direct consent or as a waived exemption. This regulation must be considered in some form any time human samples are used. This includes if the human samples are in the form of a whole slide image (WSI). |
| 21 CFR 54[3] | Financial Disclosure by Clinical Investigators | US regulation that acts to minimize a potential source of bias, which could be a financial conflict of interest by the clinical investigator(s). | Compliance must be considered when a sample testing site is acting as a clinical trial site with a designated investigator in a clinical study. |
| 21 CFR 56[4] | Institutional Review Boards | US regulation that defines the standards for the composition, operation, and responsibility of an IRB that reviews clinical investigations. | Compliance is needed for sample testing when the IDE regulation must be considered and/or applicable because the IRB review is required for clinical investigations subject to 21 CFR 812. This extends to clinical sites that might only review and/or analyze WSIs and never receive a physical sample. |
| 21 CFR 812[5] | Investigational Device Exemption | US regulation that provides procedures for the conduct of clinical investigations of devices. | Compliance is needed with relevant provisions unless a study can be defined as an exempted investigation per 21 CFR 812(c). |
| 42 CFR 493[6] | Clinical Laboratory Improvement Amendments (CLIA) | US regulation that outlines the requirements that must be adhered to for clinical laboratories that test and provide results for human samples. | Compliance is needed when a test result supports patient management for clinical trial participants. This extends to laboratories that might only review and/or analyze WSIs and never receive a physical sample. |
| 2017/746[7] | In Vitro Diagnostic Regulation (IVDR) | EU regulation that provides the framework for in vitro diagnostic medical devices, including devices for performance studies. | Compliance/conformity is needed when the use of an assay within a performance study has a medical purpose. MDCG 2022-10 is a guidance used to determine if an assay has a medical purpose.[5] |
| ICH E6(R2)[8] | Guideline for Good Clinical Practice (GCP) | Internationally recognized standard that provides an ethical and scientific quality standard for designing, conducting, recording and reporting trials that involve the participation of human subjects. Compliance/conformity is regularly expected in the US and EU for a clinical trial. | Compliance with this guideline is recommended in any context of clinical research. The use of the results and the role of the entity within a clinical trial (e.g., sponsor, investigator, CRO) would determine which principles to apply. |
| GAMP 5[9] | A Risk-based Approach to Compliant GxP Computerized Systems | A guidance document used to provide a framework of good practice that ensures computer systems are effective and of high quality, fit for intended use, and compliant with applicable regulations. | Adherence to this standard should be considered when test results from an electronic tool or system will support a clinical trial analysis. This is the primary standard to consider for primary and secondary endpoints and is highly recommended for exploratory endpoints. |
| ISO 20916[10] | Clinical performance studies using specimens from human subjects — Good study practice | An international standard that defines good study practice for the planning, design, conduct, recording and reporting of clinical performance studies carried out to assess the clinical performance and safety of IVD medical devices for regulatory purposes | Conformity to this standard can be used to meet the clinical performance study requirements for the IVDR. This standard alone does not allow for compliance, but conformity is expected. |

*This should not be considered a complete list, but a starting guide to achieve compliance. Only a select number of standards were considered for a given region. This list also does not account for the numerous region-specific guidance documents that should be considered*

*Reference list:*

[1] Title 21 – Food and Drugs Chapter 1 – Food and Drug Administration Department of Health and Human Services Subchapter A - General; Part 11 Electronic Records; Electronic Signatures.

[2] Title 21 – Food and Drugs Chapter 1 – Food and Drug Administration Department of Health and Human Services Subchapter A - General; Part 50 Protection of Human Subjects.

[3] Title 21 – Food and Drugs Chapter 1 – Food and Drug Administration Department of Health and Human Services Subchapter A - General; Part 54 Financial Disclosure by Clinical Investigators.

[4] Title 21 – Food and Drugs Chapter 1 – Food and Drug Administration Department of Health and Human Services Subchapter A - General; Part 56 Institutional Review Boards.

[5] Title 21 – Food and Drugs Chapter 1 – Food and Drug Administration Department of Health and Human Services Subchapter H - General; Part 812 Investigational Device Exemptions.

[6] Title 42 – Public Health. Chapter 4 – Centers for Medicare & Medicaid Services , Department of Health and Human Services Subchapter G – Standards and Certification; Part 493 Laboratory Requirements.

[7] Regulation (EU) 2017/46 of the European Parliament and of the Council of 5 April 2017 on in vitro diagnostic medical devices and repealing Directive 98/79/EC and Commission Decision 2010/227/EU.

[8] International Council for Harmonisation of Technical Requirements for Pharmaceuticals for Human Use (ICH). ICH Harmonised Guideline. Integrated Addendum to ICH E6(R1) Guideline for Good Clinical Practice E6(R2). Current Step 4 version dated 9 November 2016.

[9] International Society for Pharmaceutical Engineers(ISPE), GAMP5, A Risk-Based Approach to Compliant GxP Computerized Systems. Second Edition. July 2022.

[10] ISO 20916:2019, In vitro diagnostic medical devices — Clinical performance studies using specimens from human subjects — Good study practice. Edition 1.
